# Immunotherapy in Locally Advanced Cervical Carcinoma: A Narrative Review

**DOI:** 10.3390/cancers18091409

**Published:** 2026-04-29

**Authors:** Claire Meynard, Emmanuel Fardeau, Tiphaine Lambert, Sophie Guillerm, Diane Jornet, Laurent Quero, Christophe Hennequin

**Affiliations:** 1 Radiation Oncology Department, Hôpital Saint-Louis, AP-HP, Université Paris-Cité, 75010 Paris, France; claire.meynard@aphp.fr (C.M.); emmanuel.fardeau@aphp.fr (E.F.); sophie.guillerm@aphp.fr (S.G.); diane.jornet@aphp.fr (D.J.); laurent.quero@aphp.fr (L.Q.); 2 Medical Oncology Department, Hôpital Saint-Louis, AP-HP, Université Paris-Cité, 75010 Paris, France; tiphaine.lambert@aphp.fr

**Keywords:** cervical cancer, immunotherapy, radiotherapy

## Abstract

The treatment of locally advanced cervical cancer is based on concomitant cisplatin-based chemoradiotherapy followed by brachytherapy. The quality of this treatment is essential to optimize results. In particular, intensity-modulated radiotherapy followed by image-guided brachytherapy achieves 90% local control regardless of the stage of the disease. More recently, several randomized trials have changed the management of these tumors: The INTERLACE trial evaluated the benefits of neoadjuvant chemotherapy with carboplatin and paclitaxel and demonstrated a survival benefit for this approach. This trial has been the subject of considerable criticism. The KEYNOTE A.18 trial evaluated the addition of pembrolizumab to standard treatment: an improvement in overall survival was demonstrated for patients with stage III-IV disease (FIGO 2014 classification). The CALLA trial, which evaluated the addition of durvalumab, was negative. This review summarizes the biological rationale for this immunotherapy, its results, and the quality criteria for chemoradiotherapy. It describes the various trials in detail, puts their results into context, and discusses the relevance of this new treatment based on the patients’ baseline characteristics. Based on this critical analysis, patients with stage III-IVA cervical cancer (FIGO 2014 classification) should receive, in addition to standard chemoradiotherapy, concomitant treatment with pembrolizumab followed by two years of maintenance therapy.

## Simple Summary

Until now, the treatment of locally advanced cervical cancer has relied on a combination of concurrent chemotherapy and radiation therapy. Recently, it has been demonstrated that the addition of pembrolizumab improves outcomes in stage III-IVA disease. Three-year overall survival increases from 74.8% to 82.6% (HR 0.67, *p* = 0.004). Given the immunological context in which cervical cancer develops, the effect of a PD-1 inhibitor makes sense. This new, costly approach may pose challenges in low- and middle-income countries. Furthermore, high-quality chemoradiotherapy, systematically incorporating brachytherapy, remains essential. However, the combination of immunotherapy with chemoradiotherapy represents one of the most significant advances in recent years for this disease.

## 1. Introduction

Cervical cancer is the 8th most common cancer worldwide: as reported by the WHO, it is diagnosed in 703,000 women, with 373,000 deaths reported annually [1]. Eighty to 85% of this cancer occurred in low–middle income countries (LMICs) [2]. Despite effective methods for primary and secondary prevention, namely Human Papilloma Virus (HPV) vaccination and cervical screening, these tools are inequitably distributed in LMICs [3]. In 2020, the World Health Organization (WHO) launched a strategic plan to eliminate cervical cancers [4]. However, in 2024, only 31% of females had begun a vaccination program, with, again, a great heterogeneity between countries [5].

Squamous cell carcinoma (SCC), adenocarcinoma (ADC) and adenosquamous carcinoma (ADSC) are the three most common histological subtypes. Staging requires a clinical gynecologic examination and pelvic MRI, and, for nodal evaluation and distant metastasis, an 18-FDG PET-CT. It should be noted that the classification of cervical tumors has changed in recent years (Table 1).

For locally advanced stages (FIGO 2018 IIB, IIIA, IIIB, IIIC and IVA stages), treatment is based on a combination of chemotherapy and radiotherapy, with or without surgery [6]. Recently, immunotherapy, widely used in other cancer locations, has proven effective in this context.

## 2. Standard of Care Before Immunotherapy

Unlike localized stages (FIGO 2018 I to IIA), for which the treatment of choice is surgery, concomitant chemoradiotherapy followed by brachytherapy is recommended for locally advanced cervical cancer (FIGO 2018 IIB, IIIA, IIIB, IIIC and IVa stages) [6].

External beam radiation therapy and brachytherapy techniques have evolved significantly over the past few decades. Current recommendations [7], based on the EMBRACE II prospective observational study, are to use intensity-modulated external radiation therapy (IMRT) techniques with daily imaging for a dose of 45 Gy in 25 fractions of 1.8 Gy, with an integrated boost of 55 to 57.5 Gy in cases of lymphadenopathy. Brachytherapy should ideally be guided by MRI (Image-Guided Adaptive Brachytherapy: IGABT) and, depending on the extent of the tumor, combine intracavitary and interstitial brachytherapy techniques. The aim is to achieve satisfactory coverage of the high-risk target volume, corresponding to the cervix and all residual tumor after chemoradiotherapy (cumulative dose with external radiotherapy, calculated as a dose equivalent in fractions of 2 Gy, of at least 90 Gy over 90% of this volume, while respecting the dose constraints for organs at risk).

The addition of concomitant cisplatin-based chemotherapy improves overall survival outcomes [8,9,10,11,12]. A meta-analysis of data from 18 trials comparing concomitant chemoradiotherapy with radiotherapy alone confirms this benefit in overall survival (6% in 5 yr overall survival (OS): from 60 to 66%; Hazard ratio (HR): 0.81, 95% CI: 0.71–0.9, *p* < 0.001) with the addition of concomitant chemotherapy [13]. Various concomitant chemotherapy protocols have been tested, but there is a higher level of evidence for cisplatin-based regimens, which are now the standard concomitant chemotherapy treatment in the absence of contraindications.

This standard treatment provides good results in terms of local control, ranging from 89% to 98% at 5 years in the EMBRACE I study, but disease-free survival could still be improved, ranging from 47% to 76% depending on stage in EMBRACE I [14].

Strategies to intensify systemic treatments in combination with chemoradiotherapy have been, and continue to be, studied. Several trials have focused on a strategy of neoadjuvant chemotherapy prior to radiotherapy. In an older meta-analysis [15] based on data from 18 trials and 2074 patients, a survival benefit was found with the addition of first-line chemotherapy in cases of chemotherapy with cycles of 14 days or less and a cisplatin dose intensity of at least 25 mg/m^2^. However, only trials in which radiotherapy was performed without concomitant chemotherapy, which is now the current standard, were included in this meta-analysis, therefore preventing reliable conclusions about the potential benefits of neoadjuvant chemotherapy in cases of concomitant chemoradiotherapy.

A randomized phase 2 trial [16], with or without neoadjuvant chemotherapy with cisplatin and gemcitabine, found a deleterious effect of neoadjuvant chemotherapy. However, in contrast to these rather disappointing results, the INTERLACE study [17] published in 2024 is the first large, randomized study to show a benefit to neoadjuvant chemotherapy. Five hundred patients with FIGO 2008 stage IB1 SCC, ADC or ADSC of the cervix with pelvic lymph node involvement, or stage II, IIIB, or IVA, were included. It should be noted that patients with para-aortic lymph node involvement and stage IIIA (involvement of the lower third of the vagina) were excluded. Patients were randomized to standard treatment with chemoradiotherapy followed by brachytherapy, with or without neoadjuvant chemotherapy consisting of six cycles of carboplatin AUC2 and paclitaxel 80 mg/m^2^ weekly. The primary endpoints were progression-free survival and overall survival. Seventy percent of patients had stage IIB tumors, and 57% of patients had no lymph node involvement. Most patients were able to receive five cycles of cisplatin during radiotherapy (68% in the induction chemotherapy arm and 79% in the no induction chemotherapy arm). The median follow-up was 67 months. Induction chemotherapy resulted in a significant improvement in progression-free survival (72% vs. 64% at 5 years; HR 0.65 (95% CI 0.46–0.91, *p* = 0.013) and overall survival (80% vs. 72% at 5 years; HR 0.60 (95% CI 0.40–0.91, *p* = 0.015)). However, it should be noted that the quality of radiotherapy and brachytherapy is questionable, with only 40% of patients treated with IMRT and 30% with image-guided brachytherapy. Local control was poorer in the overall population of the INTERLACE trial (83%) compared with the EMBRACE study (92% at 5 years), even though the diseases were generally lower risk: 14% stage III-IV vs. 25%, 43% of patients with lymph node involvement (excluding para-aortic involvement) vs. 52% with lymph node involvement (including paraaortic involvement) in the EMBRACE study. By constructing a cohort similar to that of INTERLACE from EMBRACE data, it was shown that the benefit of neoadjuvant chemotherapy was clearly called into question, suggesting to the authors that neoadjuvant chemotherapy merely compensated for suboptimal radiotherapy [18].

Another intensification strategy that has been studied is adjuvant chemotherapy, which has not been proven effective [19]. The randomized phase 3 OUTBACK study found no benefit to adding 4 cycles of carboplatin-taxol chemotherapy after chemoradiotherapy and brachytherapy [20].

## 3. Immunological Landscape of Cervical Cancer

Cervical cancer is most often caused by infection with the human papillomavirus (HPV), particularly subtypes 16 and 18, whose genome integrates into the epithelial cells of the cervix [21]. The process of carcinogenesis involves the viral oncoproteins E5, E6, and E7 [22], which interfere with certain cell cycle proteins. The E6 protein promotes the degradation of p53 via the ubiquitin-proteasome system. Meanwhile, E7 binds to and inactivates the retinoblastoma tumor suppressor protein (pRb) [23]. This leads to a decrease in apoptosis, tumor proliferation, and chromosomal aberrations, resulting in the development of precancerous lesions and then invasive cancer.

The immune environment is an effective barrier for eliminating abnormal cells. However, in the case of malignant tumors, this surveillance system can be overwhelmed. Following infection with the HPV, keratinocytes induce a specific immune response that promotes the persistence of the infection. Thus, the presence of Tumor Infiltrating Lymphocytes (TILs) appears necessary to enable immune evasion and the initiation of the cancer process [24].

**Figure 1:**
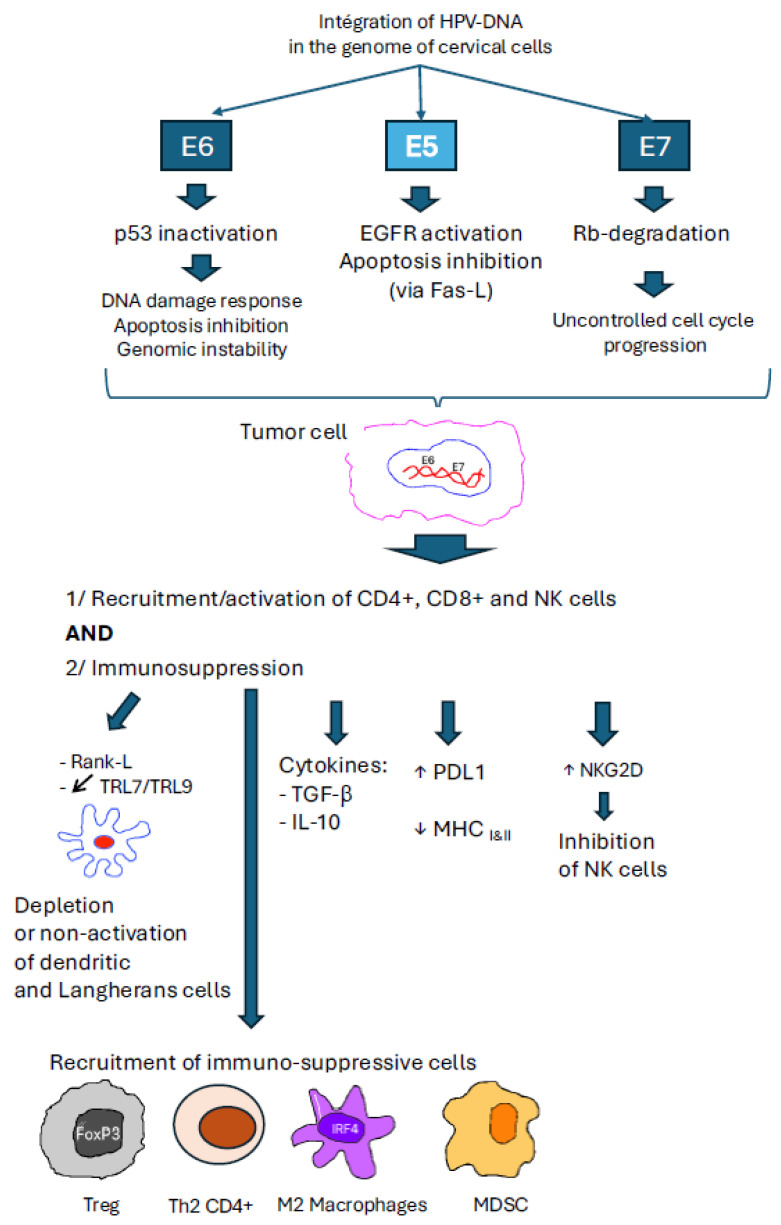
Biological and immunological consequences of HPV genome integration into cervical cells.

On one hand, E6 and E7 oncoproteins promote the recruitment and activation of immune cells; on the other hand, they induce an immunosuppressive tumor microenvironment (TME). The interplay between activation and immunosuppression explains why the tumor immune microenvironment (TME) is unique in HPV-induced cancers [25] (Figure 1).

Immune cells can be divided into immunoactive cells (Th1 T cells, NK cells and M1 macrophages) and immunosuppressive cells (Th2 T cells, B cells and tumor-associated macrophages (TAM)). Under normal conditions, the presence of CD4+ and CD8+ lymphocytes eliminates any cells undergoing transformation. Dendritic cells, which present antigens to CD4+ and CD8+ cells, appear in low concentrations in patients with chronic HPV infection [26]. This low number of dendritic cells could be explained by the secretion of Rank-Ligand (RANK-L) by tumor cells [27].

CD4+ cells are subdivided into four major subsets: regulatory T cells (Tregs), Th1, Th2 and Th9. The subtype Th1 exerts its action via different cytokines, in particular Interferon-Gamma (IFN-gamma), interleukins (IL) 2, 10, 12 and tumor necrosis factor (TNF) and facilitates the action of cytotoxic CD8+ lymphocytes. More recently, it has been shown that the Th9 subtype, via the cytokines IL9 and IL21, plays a major role in suppressing malignant transformation by inhibiting proliferation, promoting tumor cell apoptosis, and antigen unmasking through induction of major histocompatibility complex 1 (MHC-1) expression [28]. CD8+ cells are the main effectors of antitumor cytotoxicity, an effect that can be enhanced by chemotherapy [29] or immunotherapy [30]. The presence of significant CD8+ infiltration correlates with a better prognosis after chemoradiotherapy [31] and improved immunotherapy efficacy [32].

Tumor-infiltrating macrophages (TAM) also play an important role in cervical cancer. They can have an anti-tumor (M1) or a pro-tumor (M2) effect. M1-type TAMs exert an anti-tumor action either through direct cytotoxicity or via antibody-dependent cytotoxicity (ADCC). Natural killer (NK) cells are cytotoxic cells that exert their action without antigen presentation. NKG2D, a transmembrane protein of the lectin family, is overexpressed in HPV-induced tumor cells. It inhibits the function of NK cells. Other mechanisms secondary to chronic HPV infection inhibit the action of NK cells [33,34,35].

Conversely, other immune cells promote tumor growth. Treg cells have an anti-inflammatory effect but also attenuate the immune response, inhibiting the antitumor action of other immune cells. They are very frequently found in cases of chronic HPV infection [26]. Infiltration by these Treg cells correlates with the prognosis of patients treated with chemoradiotherapy [36]. Th2 cytokines (IL-4, IL-5, IL-6, IL-9, IL-10, and IL-13) promote neoplastic lesions. CD4+Th17 cells secrete a pro-inflammatory protein, IL17, which is involved in the proliferation, invasion, and angiogenesis of cervical cancers [37].

M2 macrophages promote tumor angiogenesis and epithelial–mesenchymal transition (EMT). The polarization of M1 macrophages towards the M2 phenotype is correlated with resistance to chemoradiotherapy and a poor prognosis [38]. While CD68 is a general marker for macrophages, the expression of CD163, CD23, or CD 204 indicates M2 polarization. CD68+ or CD163+ macrophages frequently appear in chronic HPV infections and are correlated with tumor progression [39,40]

The joint presence of Treg [41] and myeloid-derived suppressor cells (MDSCs) within the tumor is also a major factor in immunosuppression and is correlated with advanced stages [42]. Cancer-associated fibroblasts (CAFs), activated by tumor cells, promote tumor proliferation by creating a barrier for antitumor immune cells and through various biochemical mechanisms. In addition, they overexpress PD-L1 and PD-L2, facilitating immune evasion [43].

The viral E5 protein decreases the expression of MHC/HLA-I antigens, altering their recognition by cytotoxic T cells [44]. Finally, the E6 and E7 proteins also facilitate immune tolerance by promoting PD-L1 expression by tumor cells [45,46]. PDL1 expression levels appear to be particularly high in cervical cancers [47], but is less frequent in ADC [48]. The CPS (combined positive score) represents the ratio of the number of cells expressing PD-L1, including both tumor cells and immune cells in the tumor microenvironment. The percentage of patients that were PD-L1 positive ranged from 33% to 87% in SSC, and from 0% to 67% in ADC [49].

Overall, the TME plays a pivotal role in tumor initiation, invasion, and progression in cervical cancer. It is highly immunosuppressive as a result of HPV infection.

Radiotherapy induces both immunostimulation and immunosuppression [50]. It promotes antigen unmasking, MHC-1 expression, and DAMP secretion, factors that stimulate antitumor immunity. But at the same time, it induces PDL-1 expression [51] and promotes the recruitment of M2 and MDSC (Wanhainen, 2025 myeloid-derived suppressor cells). It also induces lymphopenia and myelosuppression.

Current therapeutic approaches aim to counterbalance the immunosuppressive effect of radiotherapy. Immune checkpoints (ICs) play an important role in the acquisition of immunosuppressive characteristics by the TME [52,53]. Immune checkpoint inhibitors (ICIs) work by blocking the inhibitory pathways generated by ICs and, in doing so, restoring the activity of cytotoxic T cells. This is particularly true for PD1/PDL1 inhibitors, whose efficacy has been particularly demonstrated in metastatic cervical cancers [54]. In addition to PD1/PDL1 inhibitors, other immunomodulatory pathways are currently being evaluated, such as CTLA-4 inhibitors, as well as LAG-3 inhibitors, which are also immunosuppressive and overexpressed in cervical cancers [55].

In conclusion, immune system perturbations play a pivotal role in cervical cancer progression and metastasis. Immunological treatments aim to reverse this phenomenon. Immunotherapy aims to counteract these immune responses and make standard treatment with chemoradiotherapy more effective.

## 4. Immunotherapy in Locally Advanced Cervical Cancer

Recently, immunotherapy has also proven effective in locally advanced cervical cancer. Several checkpoint inhibitors have been studied, but the only two randomized phase 3 studies published to date have involved pembrolizumab, an anti-PD-1 antibody (KEYNOTE A.18) [56,57,58], and durvalumab, an anti-PD-L1 antibody (CALLA study), with similar administration regimens [59]. The methodology, patient characteristics, and key results of these two large trials are summarized in Table 2, along with a summary of data from the INTERLACE trial mentioned above, another major trial published recently that could lead to changes in clinical practice.

In the KEYNOTE A.18 study [56], 1060 patients were randomized to receive either standard treatment with chemoradiotherapy followed by brachytherapy, or the same standard treatment with the addition of pembrolizumab concomitantly with chemoradiotherapy and as adjuvant therapy for a total duration of two years. The primary endpoints were progression-free survival and overall survival. The first interim analysis [57], with a median follow-up of 17.9 months, showed an improvement in progression-free survival (PFS at 24 months of 68% in the pembrolizumab arm vs. 57% in the placebo arm). The second interim analysis, with a median follow-up of 29.9 months, confirmed the improvement in progression-free survival (PFS at 36 months of 69.3% in the pembrolizumab arm vs. 56.9% in the placebo arm, HR 0.68 (95% CI 0.56–0.84)) and also demonstrated improved overall survival (OS at 36 months of 82.6% in the pembrolizumab arm vs. 74.8% in the placebo arm; HR 0.67 (95% CI 0.50–0.90, *p* = 0.004). The benefit was particularly marked in the subgroup of patients with FIGO stage III or IVA disease, both in terms of progression-free survival and overall survival. In fact, in the subgroup analysis, no significant benefit in PFS or OS was observed for patients with stage IB2 to IIB (FIGO 2014 classification).

Regarding PD-L1 expression, the CPS, which was not part of the study inclusion criteria, was positive (≥1) for the vast majority of patients (94%). The benefit in progression-free survival was found in the subgroup of patients with CPS ≥ 1 (HR 0.69 (95% CI 0.56–0.85)) but not significantly in the subgroup of patients with CPS < 1 (HR 0.57; 95% CI (0.19–1.71), and no conclusion could be drawn in this subgroup due to the small sample size (50 patients).

The reported side effects observed correspond to the known toxicity of checkpoint inhibitors (mainly thyroid disorders) without any increase in the toxicity of radiotherapy (particularly in terms of digestive problems).

Grade 3 treatment-related adverse events occurred in 75% of patients in the pembrolizumab–chemoradiotherapy group versus 69% in the placebo–chemoradiotherapy group. The adverse events for which there was an increased risk in the pembrolizumab–chemoradiotherapy group were immune-mediated adverse events (mainly thyroid disorders), accounting for only 4% of grade 3 or higher immune-mediated adverse events. There was no difference in the proportion of patients who discontinued treatment due to adverse events between the two groups (15% in the pembrolizumab–chemoradiotherapy group vs. 13% in the placebo–chemoradiotherapy group).

There was also no difference in terms of gastrointestinal toxicity between the two groups. The assessment of patients’ quality of life, using patient-reported outcomes, showed no negative impact from the addition of pembrolizumab to chemoradiotherapy [58]. It should be borne in mind, however, that the patients in the KEYNOTE A.18 trial were relatively young (median age 50 years [40,41,42,43,44,45,46,47,48,49,50,51,52,53,54,55,56,57,58,59]) and in excellent general health (WHO performance status 0 for 73% of them, WHO performance status 1 for the remaining 27%). Caution is therefore warranted when adding immunotherapy to concurrent chemoradiotherapy—a treatment that is already relatively intensive in itself—in more frail patients.

The KEYNOTE A.18 study is therefore the first to show a clear benefit from the addition of immunotherapy at the locally advanced stage, which represents a real breakthrough. One of the strengths of this study is the quality of the radiotherapy and brachytherapy, with the centralized evaluation of each treatment plan and the use of modern techniques (external radiotherapy performed using IMRT or VMAT for 89% of patients).

The other randomized phase 3 study that investigated the addition of immunotherapy in locally advanced cervical cancer is the CALLA study [59], with a similar design to the KEYNOTE A.18 study, but using durvalumab instead of pembrolizumab (chemoradiotherapy and brachytherapy with the addition of placebo or durvalumab for a total of 24 cycles every 4 weeks, starting at the same time as chemoradiotherapy). The inclusion criteria were similar. A total of 770 patients were randomized. With a median follow-up of 18.5 months, there was no significant difference in progression-free survival between the two arms (12-month PFS of 76% in the durvalumab arm vs. 73.3% in the placebo arm; 24-month PFS of 65.9% vs. 62.1%; *p* = 0.17). However, in a post hoc subgroup analysis, a benefit in progression-free survival was observed in the subgroup of patients with PD-L1 expression (TAP score ≥ 20%).

These two large randomized studies, published in close succession, therefore have conflicting results, with no clear explanation for that discordance. This may be due to the fact that the KEYNOTE A.18 study population consisted of patients with slightly more advanced disease overall than in the CALLA study, and immunotherapy may be more beneficial in more advanced disease. Indeed, a higher proportion of patients in the KEYNOTE A.18 study had lymph node involvement (83% vs. 74% and para-aortic lymph node involvement in 22% of patients vs. 11%). The definition of lymph node involvement was also stricter in the KEYNOTE A.18 study: at least two pelvic lymph nodes with a short axis ≥ 15 mm or hypermetabolic on PET scan, or one para-aortic lymph node with a short axis ≥ 15 mm or hypermetabolic on PET scan (vs. at least one lymph node with a short axis ≥ 10 mm in the CALLA study). The progression-free survival curves for the placebo arms are indeed slightly worse in the KEYNOTE A.18 study than in the CALLA study (24-month PFS of 57% vs. 62%). Another hypothesis to explain these conflicting results would be that anti-PD1 antibodies are more effective than anti-PD-L1 antibodies for cervical cancer. However, this hypothesis seems to be invalidated by the positive results of the BEATcc study with the addition of Atezolizumab (anti-PD-L1 antibody) to chemotherapy and Bevacizumab in metastatic situations [60].

In any case, Pembrolizumab has proven effective when used in combination with chemoradiotherapy and brachytherapy for locally advanced cervical cancer. It remains to be determined more precisely which patients should be offered this treatment.

For the time being, the FDA in the United States and the EMA in Europe have authorized the prescription of pembrolizumab in combination with chemoradiotherapy and brachytherapy for stage IIIA, IIIB, or IVA cervical cancer according to the 2014 FIGO classification, with or without pelvic and/or para-aortic lymph node involvement. The restriction of pembrolizumab use to this population, which does not take lymph node status into account, is related to the study’s inclusion criteria, which were based on the 2014 FIGO classification criteria, and to the results of the subgroup analysis, which showed a clearer benefit in the subgroup of patients with FIGO stage III or IVA cancer. However, it would also seem interesting to be able to offer it to patients with pelvic and/or para-aortic lymph node involvement, regardless of tumor extension, which corresponds to FIGO stages IIIC1 and IIIC2 of the new 2018 FIGO classification. The results of the KEYNOTE A.18 study in the subgroups of patients with or without lymph node involvement, particularly in patients IB2-IIB with positive nodes, have not been published.

Another question that could arise is that of PD-L1 expression: should the use of pembrolizumab be restricted to patients with PD-L1 expression? For the moment, the answer is no, since this was not one of the inclusion criteria for the KEYNOTE A.18 study, and there were too few patients with a CPS < 1 (50 patients, or 4.7% of the patients included) to draw any conclusions. However, it should be noted that the proportion of patients with a CPS ≥ 1 in the KEYNOTE A.18 study (94.3%) corresponds to the upper range of what is described in the literature (33 to 87%) [49]. It would therefore be interesting to study the benefit of pembrolizumab in PD-L1-negative patients in future studies. It should be noted that the methodology used to assess PD-L1 expression is not the same across all studies. In the KEYNOTE A.18 trial, PD-L1 expression was assessed using the Combined Positive Score (CPS). The CPS is calculated using the following formula: ((Number of PD-L1+ tumor cells) + (Number of PD-L1+ immune cells))/(Total number of viable tumor cells) × 100. In the CALLA trial, PD-L1 expression was assessed using the Tumor Area Positivity (TAP) score, which corresponds to the percentage of PD-L1+ tumor cells and surrounding immune cells, divided by the tumor area. Unfortunately, the analysis of factors that may predict the efficacy of pembrolizumab omits several important factors, such as tumor mutational burden, TIL infiltration, and data on pre-radiotherapy tumor immune status.

Other checkpoint inhibitors have been studied or are currently being studied in the context of locally advanced cervical cancer. The ATEZOLACC academic trial [61], which is a randomized phase 2 trial with a design similar to the KEYNOTE A.18 and CALLA studies but with atezolizumab, a PD-L1 inhibitor, during and after chemoradiotherapy and brachytherapy, was negative. The full results have not yet been published. The NICOL trial [62] is a phase 1 study that demonstrated the safety of Nivolumab in combination with chemoradiotherapy and brachytherapy, with encouraging PFS results.

Other ongoing trials are investigating the addition of checkpoint inhibitors as neoadjuvant therapy before chemoradiotherapy. In the COLIBRI trial [63], nivolumab + ipilimumab was evaluated with a response rate of 13% after neoadjuvant treatment and 90% after the sequence was complete. In the NRG-GY017 trial, Atezolizumab was evaluated in a randomized phase I trial [64] comparing the drug administered prior to (neoadjuvant) or during (concomitant) chemoradiotherapy: expanded tumor-associated T cell receptor (TCR) clones were higher in the neoadjuvant treatment group; no safety signals were observed. Maintenance therapy was also proposed in cases of partial or complete response after chemoradiotherapy and brachytherapy (dostarlimab in the ATOMICC trial, volrustomig in the eVOLVE cervical trial, ipilimumab in the GOG 9929 trial). The optimal sequencing of immune checkpoint inhibitors and chemoradiotherapy is still unknown. In their phase 2 trial, Duska et al. [65] compared the administration of Pembrolizumab after or during chemotherapy and observed similar results in terms of PFS and OS.

Finally, other immunotherapy strategies, such as therapeutic vaccines or cell therapies, are being studied, but with less clinical data available at this time (AIM2CERV study terminated prematurely, IMMUNOCERV study ongoing).

## 5. Quality of Staging and Chemoradiotherapy Remain Essential

The use of ICIs should not obscure the fact that the quality of chemoradiotherapy is a fundamental parameter for successful treatment. It is important to recall the fundamental points of this “classical” approach.

**PET scanning is particularly useful** for obtaining an accurate staging of the disease and is recommended prior to chemoradiotherapy being performed for curative purposes (ESGO). In particular, it improves lymph node staging [66] and allows for better definition of the pelvic lymph nodes that need to be boosted during external radiotherapy. Lymph node involvement also prompts the prescription of pembrolizumab, as already mentioned.

**Chemotherapy must be administered concomitantly:** weekly cisplatin has been the most widely studied, but alternative protocols such as the combination of 5FU and mitomycin C may also be of interest in cases of renal insufficiency [67]. Weekly carboplatin, on the other hand, appears to be less effective than cisplatin [68]. A minimum of five cycles is required, as fewer cycles reduce metastasis-free survival and local control [69,70].

**External radiation should be performed using IMRT** whenever possible, as this minimizes digestive and urinary toxicity [71]. In retrospective studies, the use of IMRT, with or without PET scanning, improves recurrence-free and overall survival [72,73]. It should not exceed a dose of 45–46 Gy, which allows a higher dose to be delivered in brachytherapy. It also allows for bone marrow preservation, which reduces acute hematological toxicity [74,75]. However, while bone marrow preservation reduces the risk of neutropenia, it has no effect on lymphopenia.

**Image-guided radiotherapy** (IGRT) with daily on-board 3D imaging is recommended to ensure safe dose application.

**Brachytherapy is an essential component of treatment** [76,77]: it allows a dose to be delivered to the cervix that cannot be achieved by any external radiotherapy technique.

**Brachytherapy should be image-guided (IGABT)** if possible, which involves performing an MRI with the applicator in place. It must follow specific recommendations, such as those of the GEC-ESTRO [78,79], with a specific definition of target volumes. Several studies have shown the lower dosimetric quality of point-directed brachytherapy compared to IGABT [80]. The EMBRACE results are particularly impressive, with a five-year survival rate of 74% and local control rates of around 90%, regardless of FIGO stage. They justify the routine implementation of this technique. In a retrospective study, it improves local control and survival [81]. A minimum dose of 85 Gy over 90% of the CTH-HR is recommended, achieving local control of 95% for squamous cell carcinomas and 86% for adenocarcinomas [82]. However, this technique requires technical resources and expertise that are often unavailable in developing countries [83]. It should also be noted that in recent trials, the number of patients treated with IGABT varies greatly, which may explain some of the results obtained: OUTBACK: 29%; INTERLACE: 30%; CALLA: 62%; KEYNOTE A18: 88.2%.

**The total treatment time should be as short as possible**: including brachytherapy, it is recommended not to exceed 55 days [84]. Indeed, despite advances in systemic treatments and radiotherapy techniques, the total duration of treatment remains a major prognostic factor [85,86].

**Regular follow-up is necessary** to detect relapses as early as possible. However, the diagnosis of persistent disease should not be made too early: in EMBRACE, out of 81 patients with persistent disease at 3 months, 60 will be in complete remission at 6–9 months without further treatment [82].

## 6. Conclusions

The combination of pembrolizumab and high-quality chemoradiotherapy achieves excellent results in locally advanced cervical cancer.

In light of the results of randomized trials, the addition of pembrolizumab to chemoradiotherapy should be considered in the treatment of stage III and IVA cervical cancer (FIGO 2014 classification).

However, many unresolved questions remain. Firstly, the selection of patients who may benefit from the addition of immunotherapy at the locally advanced stage still needs to be refined. This is particularly relevant for patients with stage IB2–IIB (FIGO 2014) disease but with pelvic or lumbosacral lymph node involvement (FIGO 2018 IIIC 1 and IIIC 2) who are not currently included in the indications but were nevertheless represented in the KEYNOTE A.18 study. It would also be interesting to have more data on the efficacy of pembrolizumab in the subgroup of patients whose tumors do not express PD-L1. They were indeed very under-represented in the KEYNOTE A.18 study.

Another question that arises is the value of neoadjuvant chemotherapy and the choice between these two strategies for intensifying systemic treatment. The INTERLACE and KEYNOTE A18 trials, testing these two different strategies of systemic treatment intensification, respectively, neo-adjuvant chemotherapy and concomitant and adjuvant immunotherapy, were both published around the same time, with positive results in both. Even though the patients are not comparable in these two studies, and some limits can be noted concerning the INTERLACE trial (notably concerning the quality of radiotherapy), a question that arises with these results is the potential benefit of combining these two strategies, calling for future trials.

This new standard of treatment, concomitant chemoradiotherapy and brachytherapy, with concomitant and adjuvant immunotherapy, is expensive, particularly for LMICs. Modern external beam radiation therapy (IMRT/IGRT) requires significant investment and regular maintenance. Image-guided brachytherapy uses MRI, which is not always readily available, and requires specific training. In addition, pembrolizumab is often delivered at a high price, so its cost-effectiveness has been debated, varying according to the prices offered in each country [87,88]. Evaluation of cost-effectiveness could be done with quality-adjusted life-years (QALYs). In the USA, pembrolizumab increased cost by $257,000 and effectiveness by 1.40 QALYs. The price of pembrolizumab must decrease from $16,990 to $9190 to be cost-effective [87].

The aim of our review is to show the optimal approach for this disease and what treatment should strive for, depending on local conditions. It is important to emphasize the role of brachytherapy, which, even though it is not image-guided, is a major factor of success.

Based on the EMBRACE data, it is unlikely that new radiotherapy techniques will significantly improve local control, except perhaps for bulky tumors. However, it is important to adhere to the quality criteria for chemoradiotherapy in order to optimize it. Efforts should currently focus on controlling metastatic disease, which occurred in 14% of the patients in the EMBRACE study [89].

The use of hypofractionated radiotherapy (around 40 Gy in 15–16 fractions) would have the dual advantage of saving machine time and shortening the duration of treatment. The data are still too immature to recommend this approach at present [90].

One possible way to improve economic efficiency would be to better select patients who benefit from immunotherapy. However, there are currently no effective predictive tests. PD-L1 expression does not appear to be the ideal tool for determining who will benefit from pembrolizumab. In the KEYNOTE A.18 trial, 94% of the patients expressed PDL1 and the absolute benefit in PFS at 36 months was “only” 12.2%. Further efforts are needed to better identify predictive markers of pembrolizumab efficacy, particularly based on the tumor’s immunological characteristics: TIL infiltration, presence of immunosuppressive cells, …

Another lead for improving economic efficiency, and patients’ quality of life, would be to reduce the duration of maintenance Pembrolizumab. The duration of two years in the Keynote A18 seems rather long, which increases the cost of the treatment, and can be difficult to accept for patients. It seems, however, unlikely that an industrial study will be conducted to investigate a shorter treatment duration.

In the future, priority should be given to prognostic and predictive studies, which will make it possible to better identify patients who can be cured by chemoradiotherapy alone and those who will require not only combined immunotherapy but also intensive systemic treatment. As previously mentioned, neo-adjuvant immunotherapy or new combinations of immunotherapy are possible approaches. Other IC, such as CTLA-4 or LAG3, are also possible targets.

Finally, treatment-related morbidity remains an important challenge for today and future treatments and must be thoroughly studied in each new trial. And in fact, health systems must focus on prevention programs (vaccination, early detection), which constitute the most cost-effective approach for this disease.

## Figures and Tables

**Table 1:** Comparison of FIGO 2014 and 2018 classifications [6].

I	The Carcinoma Is Strictly Confined to the Cervix	
IA	Invasive cancer identified only microscopically.	
IA1	Measured invasion of stroma ≤ 3 mm in depth and ≤7 mm width.	Measured invasion of stroma ≤ 3 mm in depth.
IA2	Measured invasion of stroma > 3 mm and <5 mm in depth and ≤7 mm width.	Measured invasion of stroma > 3 mm and <5 mm in depth.
IB	Clinical lesions confined to the cervix, or preclinical lesions greater than stage IA.	
IB1	Clinical lesions ≤ 4 cm in size.	Clinical lesions ≤ 2 cm in size.
IB2	Clinical lesions > 4 cm in size.	Clinical lesions > 2 cm and ≤4 cm in size.
IB3		Clinical lesions > 4 cm in size.
II	The carcinoma extends beyond the uterus, but has not extended onto the pelvic wall or to the lower third of the vagina.	
IIA	Involvement of up to the upper 2/3 of the vagina. No obvious parametrial involvement.	
IIA1	Clinically visible lesion ≤ 4 cm.	
IIA2	Clinically visible lesion > 4 cm.	
IIB	Obvious parametrial involvement but not onto the pelvic sidewall.	
IIIA	Involvement of the lower vagina but no extension onto pelvic sidewall.	
IIIB	Extension onto the pelvic sidewall, or hydronephrosis/non-functioning kidney.	
IIIC		Involvement of regional lymph nodes.
IIIC 1		Regional lymph node metastasis to pelvic lymph nodes only.
IIIC2		Regional lymph node metastasis to para-aortic lymph nodes, with or without positive pelvic lymph nodes.
IVA	Spread to adjacent pelvic organs (mucosa of bladder and/or rectum).	
IV B	Spread to distant organs.	

**Table 2:** Comparison of trial design, patients’ characteristics and main results of the KEYNOTE A.18, CALLA and INTERLACE trials.

	KEYNOTE A.18 [57]	CALLA [59]	INTERLACE [17]
Treatments	Radiochemotherapy and brachytherapy + concomitant and adjuvant pembrolizumab for 2 years vs. Radiochemotherapy and brachytherapy + placebo	Radiochemotherapy and brachytherapy + concurrent and adjuvant durvalumab for 2 years vs. Radiochemotherapy and brachytherapy + placebo	Neoadjuvant chemotherapy (6 weekly cycles of carboplatin and paclitaxel) + chemoradiotherapy and brachytherapy vs. chemoradiotherapy and brachytherapy
Inclusion Criteria	-SCC, ADC or ADSC of the cervix -stage IB2 to IIB with lymph node involvement -or stage III to IVA regardless of lymph node status	-SCC, ADC or ADSC of the cervix -stage IB2 to IIB with lymph node involvement -or stage III to IVA regardless of lymph node status	-SCC, ADC or ADSC of the cervix -stage IB1 with pelvic lymph node involvement -or stage II, IIIB, or IVA,
FIGO Classification FIGO	2014	2009	2008
Lymph node involvement definition	≥2 pelvic lymph nodes with a short axis ≥ 15 mm or hypermetabolic on PET scan, Or ≥1 para-aortic lymph node with a short axis ≥ 15 mm or hypermetabolic on PET scan	≥1 lymph node with a short axis ≥10 mm	Pelvic lymph nodes only: histologically positive or PET-positive, or at least 15 mm (short axis measurement) on CT or MRI. No positive lymph nodes above the aortic bifurcation
Number of patients	1060	770	500
Primary endpoint	PFS and OS	PFS	PFS et OS
Patient characteristics	Stage IB2 to IIB: 43% Stage III to IVA: 57% Lymph node involvement: 83% Para-aortic lymph node involvement: 22%	Stage IB2 to IIB: 34% Stage III to IVA: 66% Lymph node involvement: 74%. Para-aortic lymph node involvement: 11%	Stage IB2 to IIB: 86% Stage III to IVA: 14% Lymph node involvement: 43%. Para-aortic lymph node involvement: 0%
Quality of radiotherapy	High IMRT or VMAT: 89% Image-guided brachytherapy: 87% Centralized review of treatment plans	High IMRT or VMAT: 82% Image-guided brachytherapy: 100% Centralized review of treatment plans	Low IMRT ou VMAT: 39% Image-guided brachytherapy: 30% Radiotherapy quality assurance assessment before and during participation of all centers.
Median follow-up	29.9 months	18.6 months	67 months
Results (experimental arm vs. control)	-PFS at 36 months: 69.3% vs. 56.9% HR 0.68 (95% CI 0.56–0.84) -OS at 36 months: 82.6% vs. 74.8% HR 0.67 (95% CI 0.50–0.90), *p* = 0.004	PFS at 24 months: 65.9% vs. 62.1%; *p* = 0.17	-PFS at 5 years: 72% vs. 64%; HR 0.65 (95% CI 0.46–0.91, *p* = 0.013) -OS at 5 years: 80% vs. 72%; HR 0.60 (95% CI 0.40–0.91, *p* = 0.015)

SCC: Squamous cell carcinoma, ADC: adenocarcinoma, ADSC: adeno-squamous cell carcinoma; PFS: progression-free survival; OS: overall survival; IMRT: intensity-modulated radiotherapy; VMAT: Volumetric Modulated Arc Therapy.

## Data Availability

No new data were created or analyzed in this study.
